# Enhanced heterologous protein productivity by genome reduction in *Lactococcus lactis* NZ9000

**DOI:** 10.1186/s12934-016-0616-2

**Published:** 2017-01-03

**Authors:** Duolong Zhu, Yuxin Fu, Fulu Liu, Haijin Xu, Per Erik Joakim Saris, Mingqiang Qiao

**Affiliations:** 1 Key Laboratory of Molecular Microbiology and Technology, Ministry of Education, Nankai University, Tianjin, China; 2 Department of Food and Environmental Sciences, University of Helsinki, Helsinki, Finland; 3 College of Life Sciences, Nankai University, Room 301, Tianjin, China

**Keywords:** Microbial cell factories, *Lactococcus lactis*, Chassis, Red fluorescent protein, LecC, Heterologous

## Abstract

**Background:**

The implementation of novel chassis organisms to be used as microbial cell factories in industrial applications is an intensive research field. *Lactococcus lactis*, which is one of the most extensively studied model organisms, exhibits superior ability to be used as engineered host for fermentation of desirable products. However, few studies have reported about genome reduction of *L. lactis* as a clean background for functional genomic studies and a model chassis for desirable product fermentation.

**Results:**

Four large nonessential DNA regions accounting for 2.83% in *L. lactis* NZ9000 (*L. lactis* 9 k) genome (2,530,294 bp) were deleted using the Cre-*loxP* deletion system as the first steps toward a minimized genome in this study. The mutants were compared with the parental strain in several physiological traits and evaluated as microbial cell factories for heterologous protein production (intracellular and secretory expression) with the red fluorescent protein (RFP) and the bacteriocin leucocin C (LecC) as reporters. The four mutants grew faster, yielded enhanced biomass, achieved increased adenosine triphosphate content, and diminished maintenance demands compared with the wild strain in the two media tested. In particular, *L. lactis* 9 k-4 with the largest deletion was identified as the optimum candidate host for recombinant protein production. With nisin induction, not only the transcriptional efficiency but also the production levels of the expressed reporters were approximately three- to fourfold improved compared with the wild strain. The expression of *lecC* gene controlled with strong constitutive promoters P5 and P8 in *L. lactis* 9 k-4 was also improved significantly.

**Conclusions:**

The genome-streamlined *L. lactis* 9 k-4 outcompeted the parental strain in several physiological traits assessed. Moreover, *L. lactis* 9 k-4 exhibited good properties as platform organism for protein production. In future works, the genome of *L. lactis* will be maximally reduced by using our specific design to provide an even more clean background for functional genomics studies than *L. lactis* 9 k-4 constructed in this study. Furthermore, an improved background will be potentially available for use in biotechology.

**Electronic supplementary material:**

The online version of this article (doi:10.1186/s12934-016-0616-2) contains supplementary material, which is available to authorized users.

## Background

Bacteria are commonly used as microbial cell factories for metabolic engineering and desirable product fermentation at the laboratory scale and in industrial applications **[** 1, 2 **]**. For Gram-negative bacteria, *Escherichia coli* is a key organism utilized to construct a genetically stable strain that demonstrates robust metabolic performance [3, 4]. Shen and co-workers achieved high-titer anaerobic 1-butanol synthesis in *E. coli* [5]. Moon and co-workers achieved production of glucaric acid from a synthetic pathway in recombinant *E. coli* [6]. Hashimoto and co-workers showed that the cell size and nucleoid organization of *E. coli* cells can be changed through genome reduction [7]. The minimized *E. coli* displayed some convenience as a host to express target products, but several disadvantages were observed, such as the formation of endotoxins and inclusion in intracellular protein production [8, 9]. *Pseudomonas putida* is another bacterium selected to be constructed as robust heterologous gene expression platform. The work of de Lorenzo and co-workers showed that the streamlined-genome derivatives of *P. putida* KT2440 out competed the parental strain in every industrially relevant trait assessed, and the mutants reached a recombinant protein yield with respect to biomass of up to 40% higher than that of the wild strain [10, 11]. For Gram-positive bacteria, Morimoto and co-workers reported that they deleted 874 kb (20%) of the genomic sequence in *Bacillus subtilis* MBG874, and the heterologous protein productivity was remarkably enhanced in the mutant [12]. Unthan and co-workers initiated the construction of a chassis from *Corynebacterium glutamicum* ATCC13032 by decreasing the size of the native genome. Five strains with combinatory deletions of irrelevant gene clusters were investigated (GRS22-23, 44.0 kb deleted, accounting to 1.34% of genome; GRS23-46, 215.9 kb deleted, 6.58%; GRS16-23, 165.2 kb deleted, 5.03%; GRS21-41, 215.2 kb deleted, 6.55%; GRS41-51, 108.7 kb deleted, 3.31%); among them, three potential candidates exist, namely, GRS22-23, GRS23-46, and GRS16-23, which can be used for chassis construction [13].

*Lactococcus lactis*, which is one of the most extensively studied model organisms, exhibits superior ability to be used as engineered host for fermentation of desirable products. Purification of the targeted products from *L. lactis* is very convenient because it does not produce any endotoxins, inclusions nor many unwanted products [14, 15]. Genome reduction of *L. lactis*, which is designated as a GRAS (Generally Regarded As Safe) organism, can be applicable to a model chassis for fermentation of desirable products. Many possibly identified nonessential large DNA regions should be deleted to provide an efficient background for use in biotechnology applications and a clean background for functional genomics studies.

However, few studies are available about genome reduction of *L. lactis* despite its importance. In previous reports, some genes, such as *htrA*, *clpP*, and *ybdD*, which were involved in heterologous protein production and secretion, have been analyzed in *L. lactis*. HtrA is an extracellular protease in *L. lactis* and *B. subtilis*, and this protease plays an important role in the degradation of heterologous proteins [16]. The production of heterologous proteins can be improved when HtrA is deleted. ClpP is an intracellular protease also affecting the production of heterologous proteins [17]. The *ybdD* mutant strain shows increased levels of exported proteins [18]. The multiple protease mutant strains were also reported in Laxmi’s work [19]. In his study, not only the degradation of heterologous protein was reduced, but also the levels of cell-associated protein-folding catalysts were elevated in the multiple protease mutants. The deletion of multiple protease genes in *L*. *lactis* and *B*. *subtilis* can be an important beneficial element in the construction of protein-secreting strains [20]. To date, the construction and use of genome-streamlined *L*. *lactis* as microbial cell factory remain as attractive alternative methods to improve protein expression.

In our study, four large nonessential DNA regions accounting for 2.83% of the genome, such as prophages, transposons, and related proteins, were selected and deleted with the Cre-*loxP* deletion system in *L. lactis* NZ9000 [21]. The mutants were compared with the wild strain in several physiological traits. The mutants were also evaluated as microbial cell factories for recombinant protein production (intracellular and secretory expression) with the red fluorescent protein (RFP) [22] and bacteriocin leucocin C (LecC) [23] as reporters controlled by the nisin inducible P_*nisZ*_ and strong constitutive promoters P8 and P5 [24]. The genome-streamlined mutant *L. lactis* 9 k-4 outcompeted the wild strain in several physiological traits assessed. Additionally, *L. lactis* 9 k-4 exhibited the optimal properties among mutants as host for protein production.

## Results

### Design and construction of platform *L. lactis* strain for heterologous protein production

**Fig. 1:**
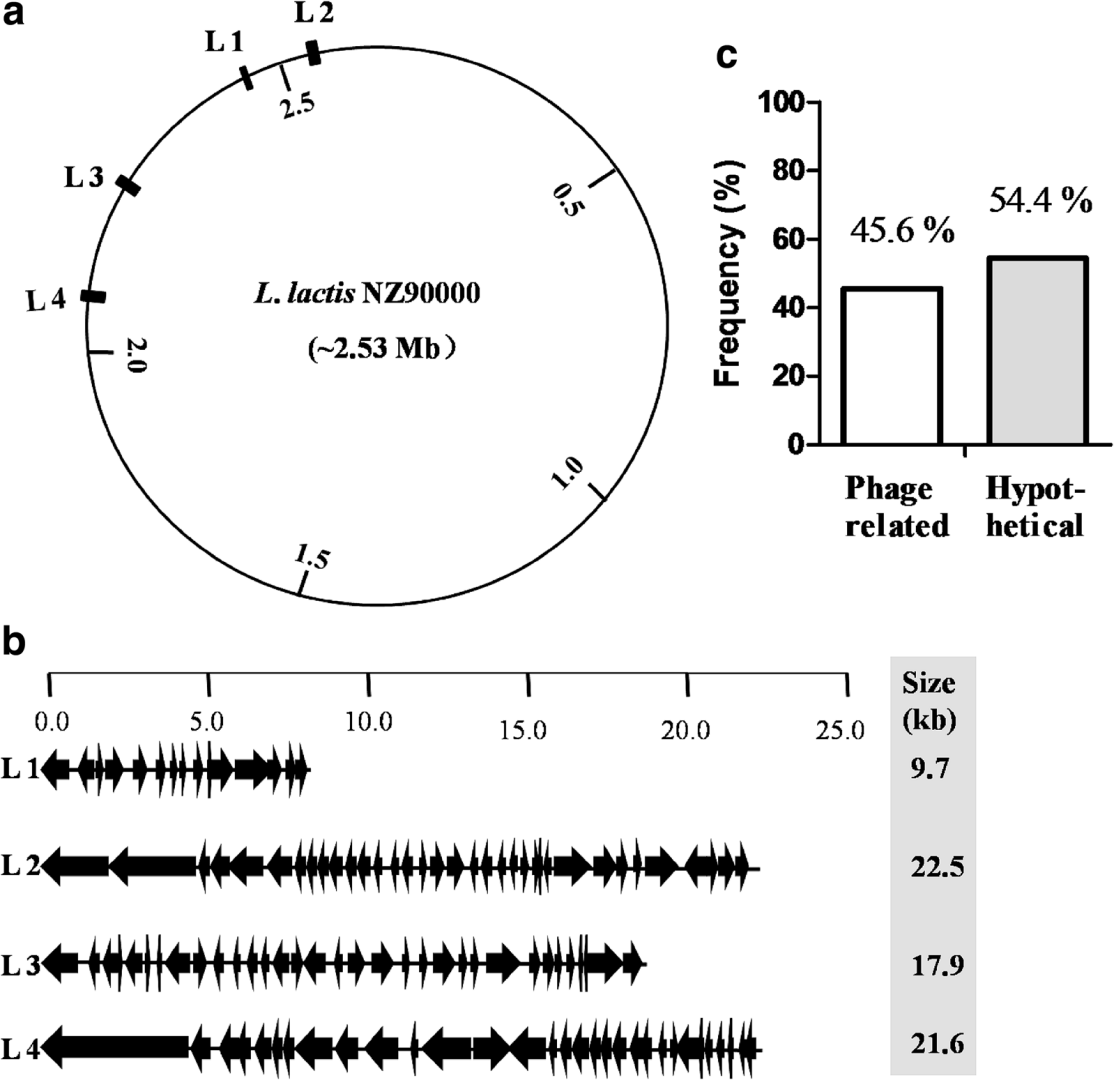
Genetic organization and deletion of four large nonessential DNA regions in *L. lactis* 9 k. **a** Circular map of *L. lactis* 9 k chromosome showing the physical location of the deletions in the genome; **b** genetic organization of four large deletions; **c** distribution and annotations of ORFs found in the large deletions

To construct the streamlined-genome mutants, we initially searched the prophage, prophage-like, and transposon genes in *L. lactis* 9 k genome sequences because prophages and transposons may not always be necessary for the strain cultured in the laboratory or industrial condition. Five prophages and related genes were identified. Four of them were successfully deleted step-by-step. The distribution of four deleted large DNA regions throughout the genome is indicated in Fig. 1a. The genetic organization of the four deleted DNA regions is shown in Fig. 1b. The first large nonessential DNA region-L1 (containing prophage 1 and related genes), which is similar to *Staphylococcus aureus* bacteriophages 80α, is 9.7 kb and contains 15 open reading frames (ORFs) [25]. Among these genes, seven have been characterized and one encodes integrase. Region L2 comprises 22.5 kb with 35 ORFs. Region L2 encodes one integrase, two transposase (IS712H)-related proteins, and five prophage ps1-related proteins. Region L3 exhibits a size of 17.9 kb and contains 34 ORFs. Eight repeat regions and six prophage-related proteins exist in L3. The phage in L3 was identified as phage Q33; Q33 is a member of the P335 species, and some of its genes encode functions such as DNA replication and packaging, morphogenesis, and host cell lysis [26]. Finally, region L4 comprises 21.6 kb, including 30 coding sequences that, except four of them, are all oriented in the same direction. Region L4 encodes 14 prophage-related proteins and three repeat regions. The phage was identified as ΦC31 through NCBI gene blast. The integrase of ΦC31 can integrate some exogenous plasmids carrying an *attB* site into native genomic sequences that bear partial identity to *attP* [27]. Detailed description of the genes is provided in Additional file 1: Table S1.

Overall, the deletion of DNA region contents of *L. lactis* 9 k comprises approximately 2.83% of its genome. Figure 1c shows the percentage of the total ORFs of four large deleted DNA regions grouped in a gross functional classification. Notably, the account reveals ∼54.4% of the corresponding ORFs encoding proteins of unknown function. The deletion results were verified by polymerase chain reaction (PCR) with testing primers (Additional file 1: Figure S1) and sequence analysis.

### Assessment of mutants’ growth parameters

**Fig. 2:**
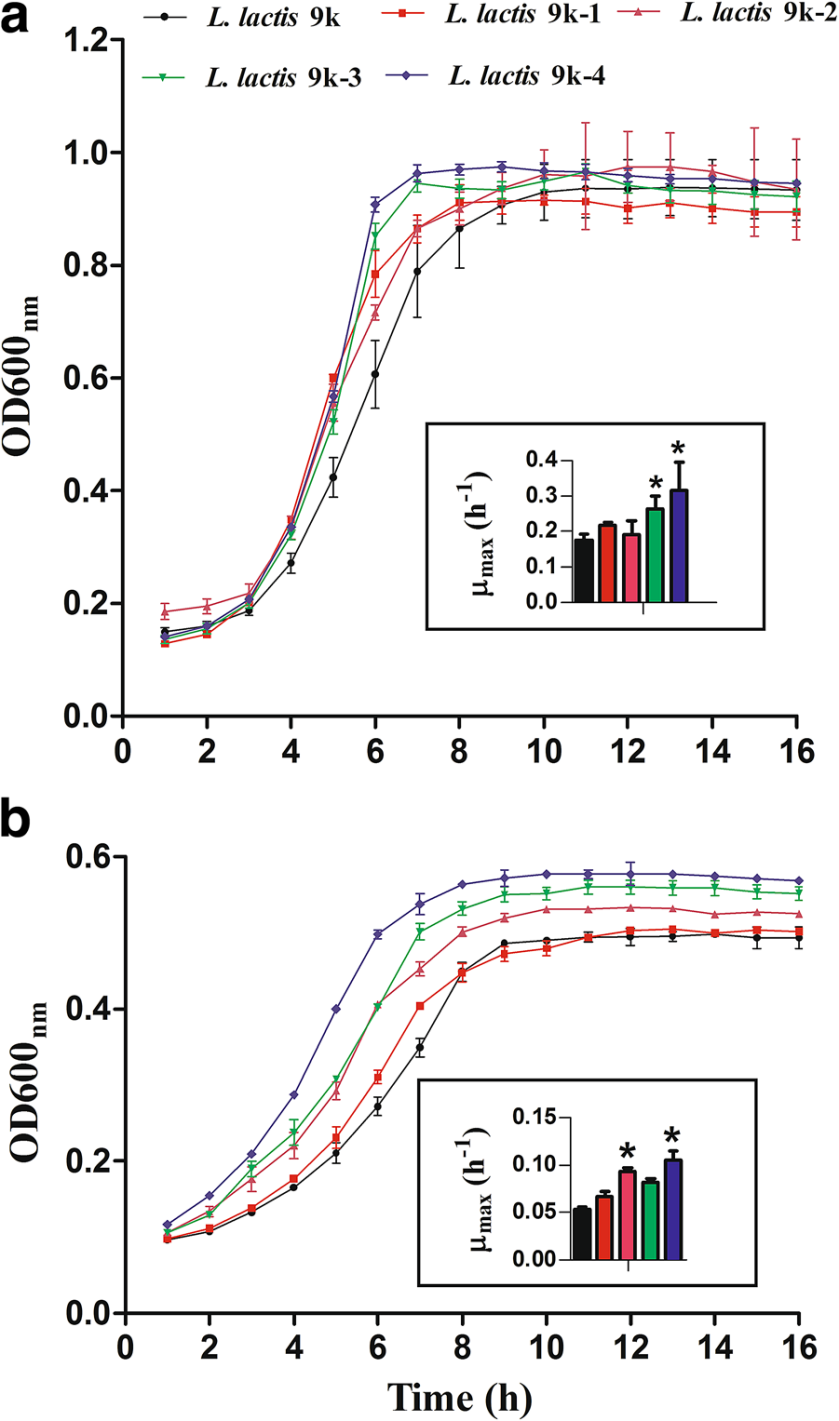
Growth profiles and maximum specific growth rate (μ_max_) of strains. The μ_max_ was determined during exponential growth. **a** Cultured in M17G media; **b** cultured in SA media. Data represent the mean of three independent experiments. *Bars* indicate standard deviations (*P < 0.05)

The growth profiles of strains were monitored by following the optical density at 600 nm (OD_600_) of cells in M17G and SA media. The growth curves are shown in Fig. 2a (M17G media) and Fig. 2b (SA media). As presented in Fig. 2a, the results showed that all the mutants started to grow exponentially 1 h before the parental strain. However, the lag growth phase of different strains exhibited no significant difference in M17G media. As shown in Fig. 2b, *L. lactis* 9 k-2, *L. lactis* 9 k-3, and *L. lactis* 9 k-4 started to grow exponentially 2 h before the wild strain when the strains were cultured in defined SA media. By contrast, *L. lactis* 9 k-1 grew similar to the wild strain. The final cell density of *L. lactis* 9 k-2 was approximately 1.2-fold higher than the wild strain, whereas the final cell densities of *L. lactis* 9 k-3 and *L. lactis* 9 k-4 were approximately 1.4-fold higher than that of the wild strain. The maximum specific growth rate (μ_max_, maximal slope of growth curve) of strains was determined during exponential growth. *L. lactis* 9 k-4 and *L. lactis* 9 k-3 showed 17 and 10% increase in μ_max_ when grown in M17G media, respectively. No significant difference was observed among *L. lactis* 9 k-1, *L. lactis* 9 k-2, and the wild strain. Interestingly, *L. lactis* 9 k-2 showed a 19% increase and *L. lactis* 9 k-4 showed a 32% increase in μ_max_ when grown in SA media. The μ_max_ difference increased between the wild strain and mutants when the strains were cultured in defined SA media.

**Fig. 3:**
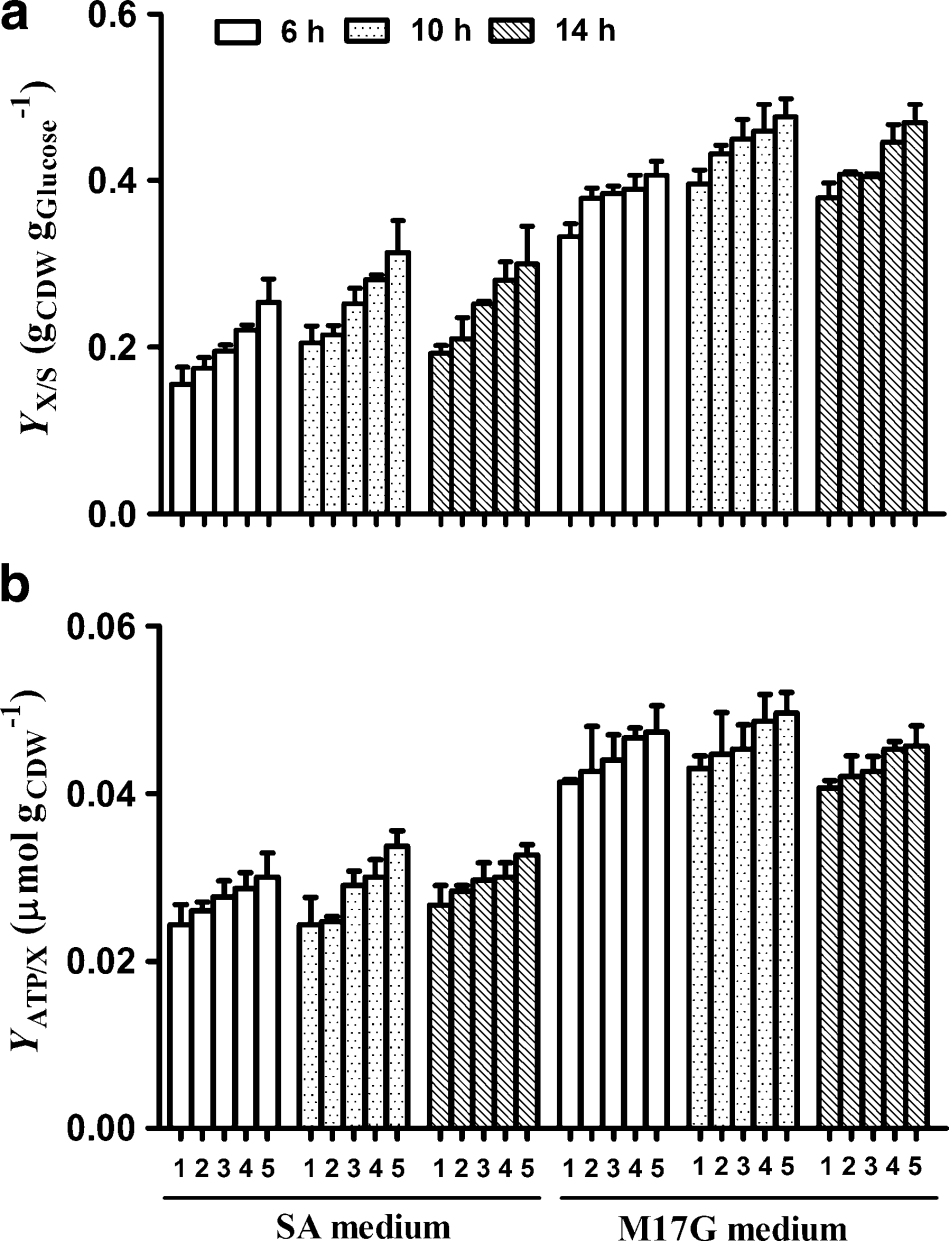
Biomass coefficient and yield of ATP on biomass. 1, *L. lactis* 9 k; 2, *L. lactis* 9 k-1; 3, *L. lactis* 9 k-2; 4, *L. lactis* 9 k-3; 5, *L. lactis* 9 k-4. **a** The biomass yield coefficient (*Y*_X/S_); **b** the yield of ATP on biomass (*Y*_ATP/X_). Data represent the mean of three independent experiments. *Bars* indicate standard deviations

All mutants attained increased final cell dry weight (CDW) on glucose (*Y*x/s, g_CDW_ g_glucose_^−1^) grown in M17G or SA media (Fig. 3a). The difference reached the maximum when the cells were cultured for 10 h in SA media. The biomass yield coefficient *Y*_X/S_ of *L. lactis* 9 k-3 was approximately 0.28 showing a 7% increase, and *L. lactis* 9 k-4 was approximately 0.32 showing a 15% increase. The energetic capacity of the cells can be estimated with the amount of adenosine triphosphate (ATP) per unit of biomass (*Y*_ATP/X_, μmol g_CDW_^−1^) [9]. Therefore, the corresponding ATP concentrations were measured. Moreover, the results were normalized to CDW to evaluate the energetic capacity of cells (Fig. 3b). The calculated results showed that *Y*_ATP/X_ of all the mutants was higher than that of the parental strain, and attained the maximum difference in SA media when grown for 10 h. In addition, *L. lactis* 9 k-3 showed a 13% increase, and *L. lactis* 9 k-4 showed a 21% increase in *Y*_ATP/X_ compared with the wild strain.

### Assessment of mutants’ phenotype

Extensive fermentation phenotype analyses of *L. lactis* 9 k-1, *L. lactis* 9 k-2, *L. lactis* 9 k-3, *L. lactis* 9 k-4, and *L. lactis* 9 k were conducted using the phenotype microarrays to understand further the physiological difference between the wild and mutant strains. All of the substrates that the mutant strains consumed were significantly different compared with the *L. lactis* 9 k, as summarized in Table 1. The results showed that *L. lactis* 9 k-2, *L. lactis* 9 k-3, and *L. lactis* 9 k-4 can efficiently metabolize nine carbon sources, particularly d-gluconic acid, maltose, d-fructose, α-d-glucose, and α-methyl-d-dgalactoside stachyose. The mutants metabolized approximately six carbon sources less efficiently. The largest deletion mutant *L. lactis* 9 k-4 exhibited good properties on some carbon source metabolism compared with the wild strain, especially for α-d-glucose and d-fructose.

**Table 1:** Phenotype assay of metabolism

Substrate consumption ratio compared to the wild strain (%)				
Substrate	*L. lactis* 9 k-1	*L. lactis* 9 k-2	*L. lactis* 9 k-3	*L. lactis* 9 k-4
l-rhamnose	10.7	12.65	22.0	34.7
Acetic acid	15.3	18.6	18.6	19.5
d-gluconic acid	−3.6	18.7	67.0	56.4
Maltose	−1.22	10.5	104.4	75.3
d-cellobiose	2.3	23.0	−1.3	74.6
d-fructose	−0.02	45.6	1.4	32.5
α-methyl-d-galactoside	2.4	1.4	41.5	28.6
Maltotriose	0.6	3.7	1.6	61.7
α-d-glucose	1.2	1.3	2.1	7.9
Stachyose	−2.2	4.2	5.1	60.1
p-hydroxy-phenylacetic acid	−22.6	−17.5	−15.5	−9.6
N-acetyl-β-d-mannosamine–N	−7.4	−9.5	−8.8	−8.4
α-Methyl-d-mannoside	−3.9	−11.3	−0.5	−9.7
d-galacturonic acid	−12.1	38.0	−9.3	−1.3
α-ketoglutaric acid	−22.1	−22.6	12.2	−1.0
d-ribose	18.2	−19.6	−8.3	−0.01

### Assessment of *lecC* gene expression in mutant strains

**Fig. 4:**
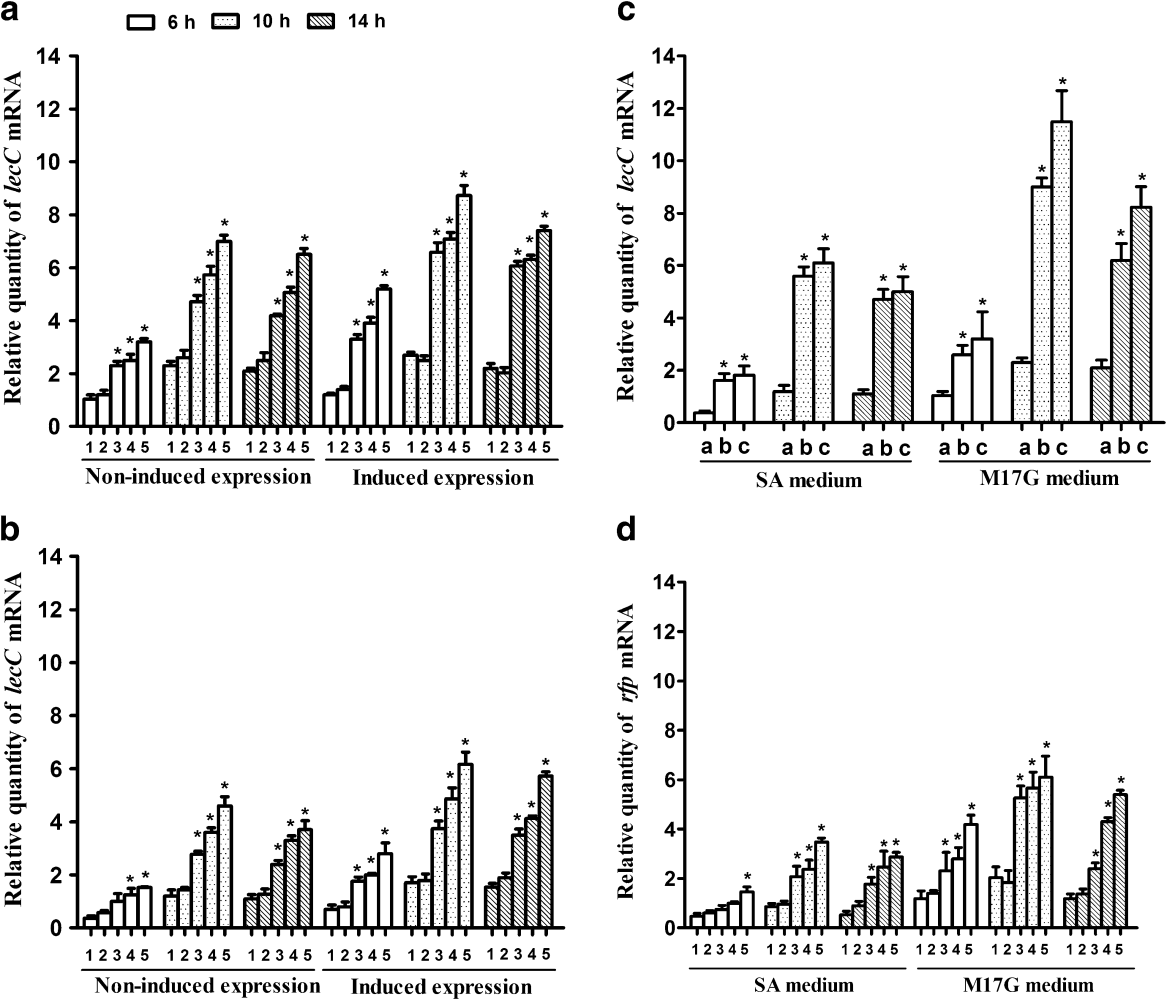
Relative quantity of *lecC* and *rfp* mRNA. 1, *L. lactis* 9 k-*lecC*; 2, *L. lactis* 9 k-1-*lecC*; 3, *L. lactis* 9 k-2-*lecC*; 4, *L. lactis* 9 k-3-*lecC*; 5, *L. lactis* 9 k-4-*lecC*; a, *L. lactis* 9 k-*lecC*; b, *L. lactis* 9 k-4-P5/*lecC*; c, *L. lactis* 9 k-4-P8/*lecC*. **a** Cultured in M17G media; **b** cultured in SA media; **c** cultured in SA or in M17G media without nisin; **d** cultured in SA or in M17G media with nisin to induce expression. Data represent the mean of three independent experiments. *Bars* indicate standard deviations (*P < 0.05)

The expression of *lecC* gene was analyzed at transcriptional and production levels through quantitative reverse transcription polymerase chain reaction (RT-qPCR) and agar-diffusion experiment. The difference of leucocin C transcriptional and production efficiency of different hosts cultured in M17G or in SA media with or without nisin to induce expression was analyzed. The results showed that the transcriptional difference of the *lecC* gene achieved the maximum when the cells were cultured for 10 h with or without nisin induction (Fig. 4a, b). The transcription of the *lecC* gene in *L. lactis* 9 k-4-*lecC*, *L. lactis* 9 k-3-*lecC*, and *L. lactis* 9 k-2-*lecC* was approximately 3.1-, 2.5-, and 2.2-fold higher than that of the wild strain, respectively, when they were cultured in M17G media with or without nisin addition for 10 h (Fig. 4a). Moreover, *L. lactis* 9 k-4-*lecC*, *L. lactis* 9 k-3-*lecC*, and *L. lactis* 9 k-2-*lecC* achieved 4.1-fold, 3.3-fold, and 2.6-fold higher transcription level of the *lecC* gene than that of the wild strain, correspondingly, when the strains were cultured in SA medium (Fig. 4b).

**Fig. 5:**
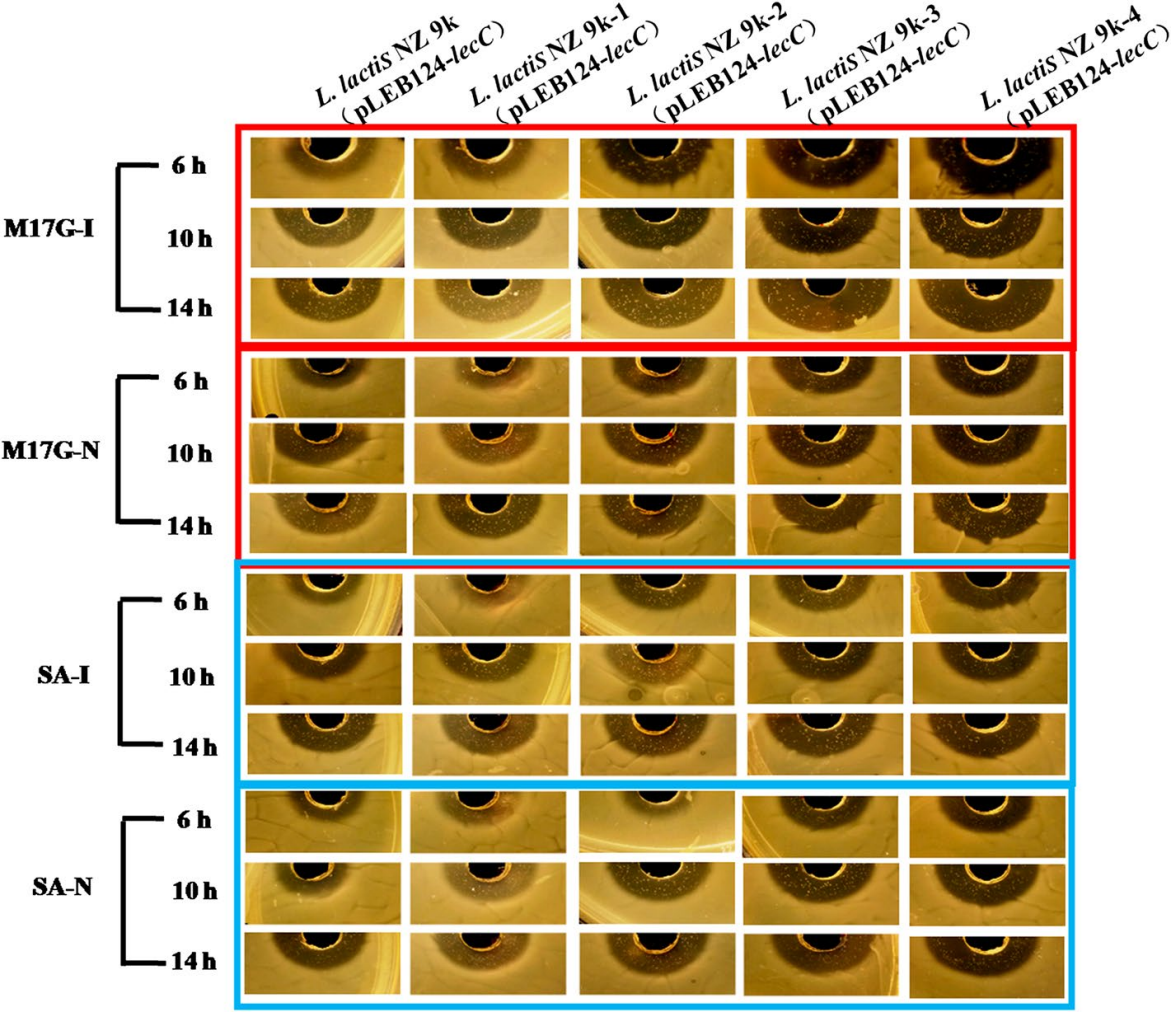
Comparison of antibacterial activity of leucocin C expressed in different mutants. *I* induced expression, *N* non-induced expression. The commercial nisin were added to the cultures at a concentration of 10 IU/ml when needed

The results of antibacterial experiment (Fig. 5) showed that the inhibiting zones of *L. lactis* 9 k-2-*lecC*, *L. lactis* 9 k-3-*lecC*, and *L. lactis* 9 k-4-*lecC* were obviously larger than that of the wild strain *L. lactis* 9 k-*lecC* regardless whether they were cultured in M17G or in SA media. When the strains were cultured for 10 and 14 h, the inhibiting zones of *L. lactis* 9 k-4-*lecC*, *L. lactis* 9 k-3-*lecC*, and *L. lactis* 9 k-2-*lecC* were approximately 2.5-, 2.0-, and 1.5-fold larger than that of the wild strain, respectively, when they were induced with nisin to express *lecC*. Growth in M17G or SA media did not influence the fold differences. Without nisin induction, the *lecC* gene was transcribed from the constitutive P45 promoter resulting in inhibition zones of 1.5-, 1.3-, and 1.2-fold larger for the *L. lactis* 9 k-4-*lecC*, 9 k-3-*lecC*, and 9 k-2-*lecC* strains than those of the wild strain, respectively. No significant difference was observed in *lecC* expression between *L. lactis* 9 k-1-*lecC* and the wild strain.

### Assessment of *lecC* gene expression under control of strong constitutive promoters in *L. lactis* 9 k-4

The strong constitutive promoters P8 and P5 were selected from the *L. lactis* promoter library and cloned in front of the *lecC* gene for expression in *L. lactis* 9 k-4. As presented in Fig. 4c, the results showed that the transcriptional efficiency of the *lecC* gene was improved significantly when it was controlled under the strong constitutive promoters P8 and P5. The transcription of *lecC* gene was improved by approximately 5.7-fold with promoter P8 and 5.0-fold with promoter P5 when the hosts were cultured in SA media. The transcription of *lecC* gene was also improved by approximately 4.8-fold with promoter P8 and 4.1-fold with promoter P5 when the hosts were cultured in M17G media.

**Fig. 6:**
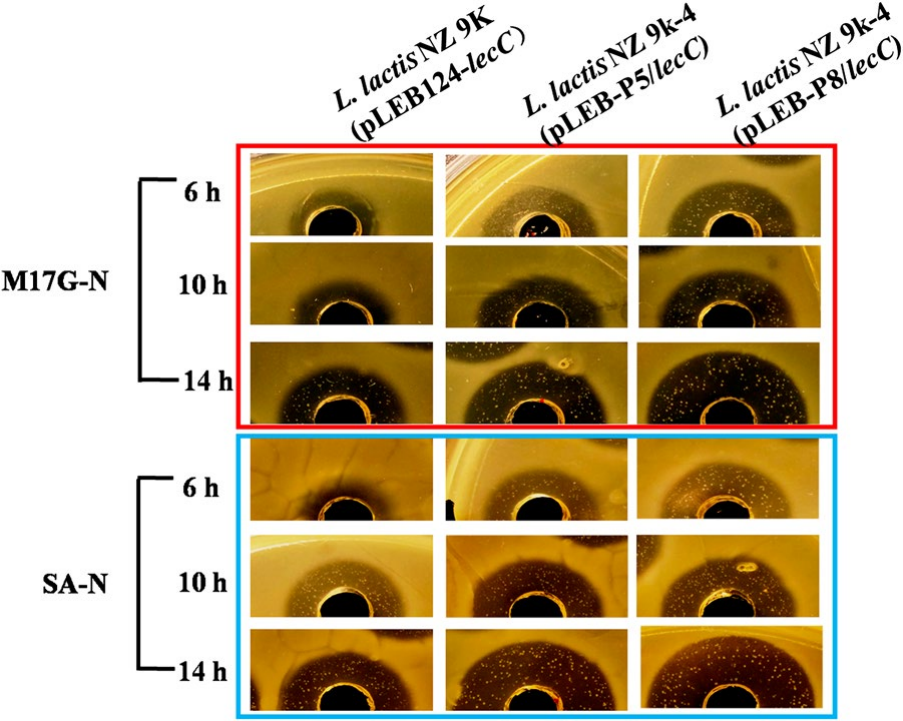
Comparison of antibacterial activity of leucocin C under control of the strong constitutive promoters P5 and P8 in the *L. lactis* 9 k-4. *N* non-induced expression

The inhibiting zones of *L. lactis* 9 k-4-P8/*lecC* and *L. lactis* 9 k-4-P5/*lecC* were obviously larger than that of *L. lactis* 9 k-*lecC* regardless whether they were cultured in M17G or SA media (Fig. 6). The inhibiting zones of *L. lactis* 9 k-4-P8/*lecC* were approximately 3.0-fold larger than that of the wild strain when the strains were cultured for 10 and 14 h. The inhibiting zones of *L. lactis* 9 k-4-P5/*lecC* were approximately 2.5-fold larger when they were non-induced to express *lecC* in M17G media. The inhibition zones of *L. lactis* 9 k-4-P8/*lecC* and *L. lactis* 9 k-4-P5/*lecC* were 2.5- and 2.0-fold larger than that of the wild strain in SA media, respectively.

### Assessment of *rfp* gene expression in mutant strains

The expression of the *rfp* gene was analyzed at transcriptional and production levels through RT-qPCR and Infinite M200 plate reader system. The transcriptional efficiency of the *rfp* reporter gene of mutant strains cultured in M17G or SA media with nisin to induce expression was analyzed (Fig. 4d). The results showed that the transcriptional difference of the *rfp* gene reached the maximum when the cells were cultured for 10 h in SA or M17G media with an inducer. The transcription of the *rfp* gene in *L. lactis* 9 k-4-*rfp* was approximately 3.0-fold higher than that of the wild strain. By contrast, the transcription of the *rfp* gene of *L. lactis* 9 k-3-*lecC* was approximately 2.2-fold higher and that of *L. lactis* 9 k-2-*lecC* was approximately 2.1-fold higher than that of the wild strain when they were cultured in M17G media for 10 h. The transcription of the *rfp* gene achieved sequentially 3.7-, 2.9-, and 2.3-fold higher than that of the wild strain when the strains were cultured in SA media.

**Fig. 7:**
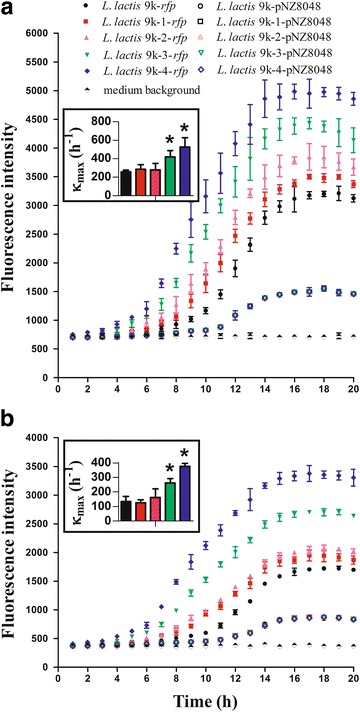
Comparison of fluorescence intensity of RFP and maximum specific rate of RFP formation (K_max_) in different mutants. K_max_ was determined during exponential growth. The strain containing plasmid pNZ8048 was used as control. **a** Cultured in M17G media; **b** cultured in SA media. Data represent the mean of three independent experiments. *Bars* indicate standard deviations (*P < 0.05)

The fluorescence intensity was traced for 20 h with Infinite M200 plate reader. This intensity reached the maximum when the cells were cultured in a 96-well plate for 17 h. The maximum specific rate (K_max_ = △RFP/△t) of RFP formation in different mutants was calculated during the exponential growth. As presented in Fig. 7, the results showed that the fluorescence intensity and K_max_ of *L. lactis* 9 k-4-*rfp* and *L. lactis* 9 k-3-*rfp* were obviously higher than that of the wild strain *L. lactis* 9 k-*lecC* regardless whether they were cultured in M17G or SA media. As shown in Fig. 7a, the fluorescence intensity of *L. lactis* 9 k-4-*rfp* was approximately 2.2-fold stronger than that of the wild strain *L. lactis* 9 k-*rfp*. By contrast, the fluorescence intensity of *L. lactis* 9 k-3-*rfp* increased by almost 1.7-fold, and that of *L. lactis* 9 k-2-*rfp* increased approximately by 1.5-fold. At the same time, *L. lactis* 9 k-4 and *L. lactis* 9 k-3 showed a 27 and 18% increase in K_max_, respectively. No statistical significant difference was observed among *L. lactis* 9 k-1, *L. lactis* 9 k-2, and wild strain when the cells were cultured in M17G media. The difference in fluorescence intensity between wild and mutant strains became large when they were cultured in SA media. As shown in Fig. 7b, the fluorescence intensity of *L. lactis* 9 k-4-*rfp* was approximately 3.1-fold stronger than that of the wild strain, whereas the fluorescence intensity of *L. lactis* 9 k-3-*rfp* was approximately 2.2-fold. Moreover, *L. lactis* 9 k-4 showed a 15% increase and *L. lactis* 9 k-3 showed a 7% increase in K_max_.

## Discussion

*L. lactis* NZ9000 has been frequently used as an expression host for nisin-induced protein production [28–30]. In this study, the genome of this host was reduced by four consecutive detetions of nonessential DNA regions with Cre-*loxP* deletion system (altogether 2.83% of the genome). The mutant strains were compared with the parental strain with regard to several physiological traits (μ_max_, K_max_, *Y*_X/S_, and *Y*_ATP/X_) and evaluated as microbial cell factories for heterologous protein production. The results showed that all mutants exhibited good characteristics in μ_max_, *Y*_X/S_, and *Y*_ATP/X_ compared with the parental strain, especially *L. lactis* 9 k-4, which can produce more CDWs and available ATPs, as well as increased μ_max_ with the same glucose consumption. Two proteins, RFP for cytoplasmic expression and bacteriocin LecC for secreted expression, were expressed in the deletion strains to test if the decreased metabolic burden would enable the host to produce efficiently heterologous proteins. These proteins were also compared with the expression in the wild strain. The results showed that decreasing the genome size yielded hosts with an increased capacity to express heterologous proteins into the cytoplasm and secreted to the medium. *L. lactis* 9 k-1 with the least decreased genome size presented the lowest effect on protein production. Increasing the genome deletion enhances the strain to perform as production host. This phenomenon was also reflected similarly in how well the cells grew in SA media and at the transcriptional level analyzed by RT-qPCR. In particular, *L. lactis* 9 k-4 with the largest deletions produced most RFP and LecC. Moreover, *L. lactis* 9 k-4 achieved the highest cell densities and was selected as host to further optimize the production plasmid from nisin-induced expression to constitutive expression with promoters P5 and P8. Constitutive expression can become more favorable than the induced expression as no inducer needs to be added; thus, production costs will be reduced [31]. The results with reporters *rfp* and *lecC* showed that with both P5- and P8-derived expression levels, the host *L. lactis* 9 k-4 can more efficiently produce the reporters than with nisin-induced production. This finding exemplifies that the *L. lactis* 9 k-4 host with its decreased genome size is a good candidate host for protein production either for cytoplasmic or secreted production with nisin-induced or constitutive expression.

To our knowledge, any unnecessary gene product that is expressed in a production host represents a potential contaminant that can drive up the cost of product purification [32]. Protein purification from spent minimal medium is easy because such media are defined and exhibiting decreased amounts of colored substances that may influence the purification proceses. As such, the genome-streamlined mutant *L. lactis* 9 k-4 is a promising foundation for further biotechnological applications.

## Conclusions

To our knowledge, this study is the first report that determined the contribution of genome reduction on the heterologous protein production in *L*. *lactis*. The genome-streamlined *L. lactis* was constructed as we expected. In addition, some mutants exhibited good properties as hosts for protein production. This finding indicated that the process of introducing further rational deletions based on transcriptome data, gene function information, and comparative genomics to construct simple predictable *L. lactis* platform for industrial use in future work, according to our specific design, is worthwhile and can contribute to expand the use of *L. lactis*.

## Methods

### Bacterial strains, plasmids, and culture conditions

The strains and plasmids used in this work are listed in Table 2. Both *L. lactis* NZ9000 (*L. lactis* 9 k) and its derivatives were grown at 30 °C under static condition in M17G media supplemented with 0.5% (w/v) glucose or in defined SA medium supplemented with 0.5% (w/v) glucose [39]. Solid medium contained 1.5% agar. *E. coli* DH5α was used as intermediate cloning host and was grown aerobically at 37 °C in Luria–Bertani broth (1% tryptone, 0.5% yeast extract, and 1% NaCl). *L. lactis* 9 k was also used as intermediate cloning host when the plasmid pNZ8048 was used to express the RFP. *Listeria monocytogenes* WSLC 1019 was cultured aerobically in Brain Heart Infusion (BHI) broth (Sigma, St. Louis, USA) at 37 °C and was used as indicator strain to detect the antibacterial activity of leucocin C. Antibiotic selection was used when appropriate: for *E. coli* (in milliliters), 150 μg of erythromycin and 15 μg of chloramphenicol; and for *L. lactis* (in milliliters), 5 μg of erythromycin and 5 μg of chloramphenicol.

**Table 2:** Bacterial strains and plasmids utilized in this study

Item	Genotype or phenotype	Reference
Strains		
*E. coli* DH5α	Cloning host; F-φ80*lacZ*△M15*endA1 recA1 endA1 hsdR17* (rK-mK+) *supE44 thi*-*1 gyrA 96 relA1* △ (*lacZYA*-*argF*)*U169 deoR λ* ^−^	[33]
*L. monocytogenes* WSLC 1019	Animal isolate (ATCC 1916), used as indicator strain	[34]
*L. lactis* N8	Constitutive strong promoters obtained from this strain	[35]
*L. lactis* NZ9000 (9 k)	MG1363 *pepN*::*nisRK*, the original strain	[36]
*L. lactis* 9 k-1	The first DNA region L1 (about 9.7 kb) deletion in *L. lactis* 9 k	This work
*L. lactis* 9 k-2	The L2 (about 22.5 kb) deletion in *L. lactis* 9 k-1	This work
*L. lactis* 9 k-3	The L3 (about 22.2 kb) deletion in *L. lactis* 9 k-2	This work
*L. lactis* 9 k-4	The L4 (about 21.6 kb) deletion in *L. lactis* 9 k-3	This work
*L. lactis* 9 k-*lecC*	Em^r^, *L. lactis* 9 k derivative containing pLEB124-*lecC*	This work
*L. lactis* 9 k-1-*lecC*	Em^r^, *L. lactis* 9 k-1 derivative containing pLEB124-*lecC*	This work
*L. lactis* 9 k-2-*lecC*	Em^r^, *L. lactis* 9 k-2 derivative containing pLEB124-*lecC*	This work
*L. lactis* 9 k-3-*lecC*	Em^r^, *L. lactis* 9 k-3 derivative containing pLEB124-*lecC*	This work
*L. lactis* 9 k-4-*lecC*	Em^r^, *L. lactis* 9 k-4 derivative containing pLEB124-*lecC*	This work
*L. lactis* 9 k-4-P8/*lecC*	Em^r^, *L. lactis* 9 k-4 derivative containing pLEB124-P8/*lecC*	This work
*L. lactis* 9 k-4-P5/*lecC*	Em^r^, *L. lactis* 9 k-4 derivative containing pLEB124-P5/*lecC*	This work
*L. lactis* 9 k-*rfp*	Cm^r^, *L. lactis* 9 k derivative containing pNZ8048-*rfp*	This work
*L. lactis* 9 k-1-*rfp*	Cm^r^, *L. lactis* 9 k-1 derivative containing pNZ8048-*rfp*	This work
*L. lactis* 9 k-2-*rfp*	Cm^r^, *L. lactis* 9 k-2 derivative containing pNZ8048-*rfp*	This work
*L. lactis* 9 k-3-*rfp*	Cm^r^, *L. lactis* 9 k-3 derivative containing pNZ8048-*rfp*	This work
*L. lactis* 9 k-4-*rfp*	Cm^r^, *L. lactis* 9 k-4 derivative containing pNZ8048-*rfp*	This work
Plasmids		
pNZ5319	Cm^r^, Em^r^, used as knock-out vector	[37]
pNZ5319△L1	Cm^r^, Em^r^, the first DNA region L1 knock-out vector	This work
NZ5319△L2	Cm^r^, Em^r^, L2 knock-out vector	This work
pNZ5319△L3	Cm^r^, Em^r^, L3 knock-out vector	This work
pNZ5319△L4	Cm^r^, Em^r^, L4 knock-out vector	This work
pLEB124	Em^r^, containing strong constitutive promoter P45	[38]
pEB690-*lecC*	Nisin^r^, containing the P*nisZ*+Usp45-*lecC*+*lecI* fragment	[23]
pLEB124-*lecC*	Em^r^, pLEB124 derivative containing the P45+P*nisZ*+Usp45-*lecC*+*lecI* fragment	This work
pLEB124-P8/*lecC*	Em^r^, pLEB124 derivative containing the P8+P*nisZ*+Usp45-*lecC*+*lecI* fragment	This work
pLEB124-P5/*lecC*	Em^r^, pLEB124 derivative containing the P5+P*nisZ*+Usp45-*lecC*+*lecI* fragment	This work
pNZ8048	Cm^r^	[36]
pET-28a-*rfp*	Kan^r^, containing *rfp* gene	Aviva systems biology
pNZ8048-*rfp*	Cm^r^, pNZ8048 derivative containing the *rfp* gene	This work

### DNA manipulations and cloning

DNA markers, T4 DNA ligase, restriction enzymes, and DNA gel extraction kit were purchased from Takara (Dalian, China). PCR product purification kit, First-strand cDNA synthesis kit, and SYBR Green RT-qPCR kit were purchased from Thermo Fisher Scientific (Thermo Fisher Scientific, Waltham, USA). The commercial nisin was purchased from Sigma (St. Louis, USA). *L*. *lactis* plasmid DNA, chromosomal DNA, and total RNA were isolated using a Qiaprep spin kit (small scale) following the manufacturer’s instructions. PCRs were performed with the Phusion enzyme (Finnzymes, Espoo, Finland). Primers used in this study are listed in the Additional file 1: Table S2. Primers were purchased from BGI (Beijing, China). Recombinant plasmids were introduced into *L. lactis* by electrotransformation as previously described [40]. Electrotransformation was performed using a Bio-Rad Gene Pulser (Bio-Rad Laboratories, Richmond, USA). *E*. *coli* DH5α was transformed through the CaCl_2_ procedure [41].

### Deletion of large nonessential DNA regions

The sequence of *L. lactis* 9 k genome was searched and checked on the NCBI website (http://www.ncbi.nlm.nih.gov/nuccore/389853198?report=graph). Several hypothetical proteins and prophage-related proteins were found. Among these genes, five prophages and related proteins (large nonessential DNA regions) were identified, and four large nonessential DNA regions were deleted on schedule with the Cre-*loxP* system by using the method described previously by our group [21]. The gene knockout vectors pNZ5319△L1, pNZ5319△L2, pNZ5319△L3, and pNZ5319△L4 were constructed subsequently. The first nonessential region was deleted in *L. lactis* 9 k with the plasmid pNZ5319△L1. The derivative was named as *L. lactis* 9 k-1. The second nonessential region was then deleted in *L. lactis* 9 k-1 with the plasmid pNZ5319△L2. The derivative was named *L. lactis* 9 k-2. The deletion step was repeated with pNZ5319△L3 and pNZ5319△L4 plasmids. The resulting mutants were named *L. lactis* 9 k-3 and *L. lactis* 9 k-4, respectively. The correct deletions of the four constructed strains were confirmed by PCR and nucleotide sequence analysis.

### Evaluation of physiological profiles

*Lactococcus lactis* 9 k, *L. lactis* 9 k-1, *L. lactis* 9 k-2, *L. lactis* 9 k-3, and *L. lactis* 9 k-4 were cultured to OD_600_ of 0.8 in the M17G medium and diluted to an optical density of OD_600_ of 0.2. Afterward, 20 μl cultures were incubated into 200 μl M17G and SA media in a 96-well plate. The corresponding bacteria were also incubated into the 100 ml of media in shake flasks. The growth profiles were monitored by measuring OD_600_ for 16 h at 30 °C by using a Bioscreen machine (Lab-systems, Helsinki, Finland) [23]. Ten milliliter suspension from shake flask was centrifuged when the cells were cultured for 6, 10, and 14 h, and the CDW was calculated using the previously described method [11]. The residual glucose in different strains was detected using commercial kits following the manufacturers’ instructions (Thermo Fisher Scientific, Waltham, USA). The ATP concentration was determined through bioluminescence assay with recombinant firefly luciferase and its substrate d-luciferin by using the Molecular Probes’ ATP Determination Kit (Thermo Fisher Scientific, Waltham, USA). One milliliter cultures of different strains were centrifuged at 6000×*g* for 5 min before the measurement of ATP concentration. Subsequently, 2 ml of 0.015 g/ml trichloroacetic acid (TCA) was added to the cells and vortexed for 3 min to extract ATP from cells. Afterward, 1 ml of supernatant from vortex was collected and diluted to the concentration of TCA lower than 0.001 g/ml with Tris–acetate buffer (PH 7.8). The extrication of ATP can be used for ATP concentration detection [42, 43]. Each sample was analyzed in triplicate.

### Evaluation of phenotype

The metabolism of wild and mutant strains was identified with GP2 MicroPlate™ by the phenotype microarray system (Biolog, California, USA). Sample preparation and assays were conducted in accordance with the manufacturer’s instructions. Briefly, the fresh cultured cells of lactococcal strains on the surface of the solid medium were collected by cotton swab and then dissolved into inoculating fluid (0.40% sodium chloride, 0.03% Pluronic F-68, and 0.02% Gellan Gum) (Biolog, California, USA). Cell density of different strains was equalized to maintain the same number of incubated bacteria. One hundred and fifty microliter tested samples were then pipetted into GP2 plate with various substrates. The plates with the sealing were incubated in the OmniLog® instrument (Biolog, California, USA) at 30 °C for 24 h. The machine automatically recorded the test data once per 30 min. The plates were shaken for 15 s before the data were recorded. Up to two strains can be tested and compared per experiment. Data were analyzed using OL-OM software (version 3.0) (Biolog, California, USA).

### Construction of plasmids with red fluorescent protein (*rfp*) reporter and leucocin C (*lecC*) reporter

The *rfp* gene was amplified with pET-28a-*rfp* plasmid as the template by using the primer pair P*rfp*-F/P*rfp*-R. The PCR product *rfp* gene fragment was digested with *Kpn*I and *Xba*I and cloned into *Kpn*I-*Xba*I digested pNZ8048, yielding pNZ8048-*rfp* plasmid.

The plasmid pLEB690-*lecC* [19] was digested with *Hin*dIII and *Bam*HI. The fragment (P*nisZ*+Usp45-*lecC*+*lecI*) was obtained with DNA gel extraction kit and was cloned into *Hin*dIII-*Bam*HI digested pLEB124, yielding pLEB124-*lecC* (containing the fragment P45+P*nisZ*+Usp45-*lecC*+*lecI*). The strong constitutive promoters P8 and P5 were amplified with the *L. lactis* N8 chromosomal DNA as the template by using the primer pairs P8-F/R and P5-F/R. P8 and P5 were also cloned into *Hin*dIII-*Bgl*II digested pLEB124-*lecC* yielding pLEB124-P8/*lecC* (containing the fragment P8+P*nisZ*+Usp45-*lecC*+*lecI*) and pLEB124-P5/*lecC* (containing the fragment P5+P*nisZ*+Usp45-*lecC*+*lecI*), respectively.

The recombinant plasmids pNZ8048-*rfp* and pLEB124-*lecC* and control plasmids pNZ8048 and pLEB124 were introduced into *L. lactis* 9 k, *L. lactis* 9 k-1, *L. lactis* 9 k-2, *L. lactis* 9 k-3, and *L. lactis* 9 k-4, respectively. The plasmids pLEB124-P8/*lecC* and pLEB124-P5/*lecC* were also transformed into *L. lactis* 9 k-4, respectively. The hosts containing these recombinant plasmids were named as *L. lactis* 9 k-*lecC* (or *rfp*), *L. lactis* 9 k-1-*lecC* (or *rfp*), *L. lactis* 9 k-2-*lecC* (or *rfp*), *L. lactis* 9 k-3-*lecC* (or *rfp*), *L. lactis* 9 k-4-*lecC* (or *rfp*), *L. lactis* 9 k-4-P8/*lecC*, and *L. lactis* 9 k-4-P5/*lecC*. The commercial nisin was added to the cultures at a concentration of 10 IU/ml to induce *lecC* expression when the cells were cultured for 5 h [38].

### Evaluation of transcriptional efficiency of the *rfp* and the *lecC* reporter

The total RNA of different hosts with the same cell density through dilution of mutant strains was extracted from the cells cultured for 6, 10, and 14 h, correspondingly. The transcription of *rfp* and *lecC* genes was then analyzed through RT-qPCR with the primer pairs Q-*rfp*-F/R and Q-*lecC*-F/R by using the comparative CT (2^−△△CT^) method with *L. lactis tufA* gene [44]. *L. lactis tufA* is a housekeeping gene coding for elongation factor Tu required for the continued translation of mRNA as a control with the primer pair *tufA*-F/R. All RT-qPCRs were repeated thrice, independently. In addition, a transcription with more than twofold change was regarded as statistically significant [45].

### Evaluation of leucocin C antibacterial activity and RFP expression

The antibacterial activity of leucocin C was determined by conventional agar-diffusion method [46]. The indicator strain *L. monocytogenes* WSLC 1019 was cultured to OD_600_ of 0.7 in BHI broth. Eight hundred microliters of indicator cultures were added to 100 ml of 60 °C BHI agar media to produce indicator plates. *L. lactis* 9 k-*lecC*, *L. lactis* 9 k-1-*lecC*, *L. lactis* 9 k-2-*lecC*, *L. lactis* 9 k-3-*lecC*, *L. lactis* 9 k-4-*lecC*, *L. lactis* 9 k-4-P8/*lecC*, and *L. lactis* 9 k-4-P5/*lecC* were incubated into M17G and SA media. Up to 5 ml cultures of different strains were collected when the cells were cultured for 6, 10, and 14 h. The cell density of mutants was diluted at similar density of the wild strain culture. Supernatants were then collected by centrifugation at 12,000×*g* for 10 min and were incubated at 70 °C for 15 min. Afterward, 50 μl of supernatants was used in the agar-diffusion experiment. The indicator plates were cultured at 37 °C for 24 h. The expression difference of *lecC* with nisin to induce expression in different hosts was also analyzed with addition of the commercial nisin to the cultures at a concentration of 10 IU/ml after the cells had been cultured for 5 h. The indicator plates and agar-diffusion experiment were prepared as described above.

*L. lactis* 9 k-*rfp*, *L. lactis* 9 k-1-*rfp*, *L. lactis* 9 k-2-*rfp*, *L. lactis* 9 k-3-*rfp*, and *L. lactis* 9 k-4-*rfp* were incubated into M17G and SA medium with 96-well plates as described above. The commercial nisin was added to the cultures at a concentration of 2 IU/ml to induce *rfp* gene expression. The cell density of strains (OD_600_) and the fluorescence intensity of RFP (excitation: 587 nm; emission: 610 nm) were monitored once every hour by Infinite M200 plate reader (Lab-systems, Helsinki, Finland) [47]. Each sample was analyzed in triplicate. The maximum specific rate (K_max_ = △RFP∕△t) of RFP formation in different mutants was calculated during the exponential growth. The difference quotient method was used to calculate the slope of RFP formation. The values of fluorescence intensity at 6, 7, 8 and 9 h were used to calculate the slope of *L. lactis* 9 k-3-*rfp* and *L. lactis* 9 k-4-*rfp* [K_max_ = (△RFP_7–6_ + △RFP_8–7_ + △RFP_9–8_)∕3], respectively, while the values of fluorescence intensity at 8, 9, 10 and 11 h were used to calculate the slope of *L. lactis* 9 k-*rfp*, *L. lactis* 9 k-1-*rfp* and *L. lactis* 9 k-2-*rfp* [K_max_ = (△RFP_9–8_ + △RFP_10–9_ + △RFP_11–10_)∕3], respectively.

### Statistical analysis

The reported experiments were independently repeated at least three times (as indicated in the text). Data were shown as mean ± standard deviation (SD). All continuous cultivations were carried out in independent biological triplicates, and each sample was additionally taken in technical triplicates. The difference between two groups was compared by *t*-test defining a *P* value <0.05 as significant.

## Additional file

**Additional file 1: Figure S1.** Verification of deletions with testing primer pairs. **Table S1.** Gene content of deleted DNA regions. **Table S2.** Primers utilized in this study.

## Abbreviations

RFP: red fluorescent protein
LecC: bacteriocin leucocin C
CDW: cell dry weight
Four large nonessential DNA regions: L-1, L-2, L-3, L-4
