# Supplementary material for: Enhanced heterologous protein productivity by genome reduction in Lactococcus lactis NZ9000
Source: Microb Cell Fact. 2017 Jan 3;16:1. doi: 10.1186/s12934-016-0616-2 (PMC5210298; doi:10.1186/s12934-016-0616-2)
Supplement: Supplementary file 1 — Additional file 1: Figure S1. Verification of deletions with testing primer pairs. Table S1. Gene content of deleted DNA regions. Table S2. Primers utilized in this study. [file 12934_2016_616_MOESM1_ESM.docx]

**Table S1. Gene content of deleted DNA regions**

| **Item** | **Gene location** | **Note** |
| --- | --- | --- |
| **The first deleted DNA region (2482702-2492442, 9.7 kb, 15 ORFs) and flanking genes** | | |
| 1 (up gene) | complement (2482328..2482702) | /note="COG2824 Uncharacterized  Zn-ribbon-containing protein involved in  phosphonate metabolism"  /product="putative alkylphosphonate uptake  protein" |
| 2 (1178 bp) | complement (2482743..2483921) | /note="COG0582 Integrase"  /product="putative integrase" |
| 3 (602 bp) | complement (2484449..2485051) | /note="COG3655 Predicted transcriptional regulator"  /product="hypothetical protein" |
| 4 (203 bp) | 2485213..2485416 | /note="COG3655 Predicted transcriptional regulator"  /product="SaPI1 Orf21" |
| 5 (692 bp) | 2485435..2486127 | /note="COG3646 Uncharacterized phage-encoded protein"  /product="hypothetical protein" |
| 6 (533 bp) | 2486381..2486914 | /product="hypothetical protein" |
| 7 (281 bp) | 2486907..2487188 | /product="hypothetical protein" |
| 8 (239 bp) | 2487231..2487470 | /product="hypothetical protein" |
| 9 (254 bp) | 2487467..2487721 | /note="COG0574 Phosphoenolpyruvate  synthase/pyruvate phosphate dikinase"  /product="hypothetical protein" |
| 10 (329 bp) | 2487718..2488047 | /product="hypothetical protein" |
| 11 (107 bp) | 2488052..2488159 | /product="hypothetical protein" |
| 12 (794 bp) | 2488156..2488950 | /product="hypothetical protein" |
| 13 (1628 bp) | 2488961..2490589 | /note="COG3378 Predicted ATPase"  /product="hypothetical protein" |
| 14 (542 bp) | 2490817..2491359 | /product="hypothetical protein" |
| 15 (302 bp) | 2491632..2491934 | /product="hypothetical protein" |
| 16 (410 bp) | 2492008..2492418 | /product="hypothetical protein" |
| 17 (down gene) | 2492442..2492765 | /note="COG1595 DNA-directed RNA polymerase  specialized sigma subunit, sigma24 homolog"  /product="hypothetical protein" |
| **The second deleted DNA region (32472-54990, 22.5 kb, 35 ORFs) and flanking gene** | | |
| 1 (up gene) | 30938..32104 | /note="COG0246 Mannitol-1-phosphate/altronate dehydrogenases"  /product="mannitol-1-phosphate 5-dehydrogenase" |
| 2 (323 bp) | complement (32974..33297) | /product="hypothetical protein" |
| 3 (380 bp) | complement (33398..33778) | /note="COG1595 DNA-directed RNA polymerase  specialized sigma subunit, sigma24 homolog"  /product="hypothetical protein" |
| 4 (302 bp) | complement (33806..34108) | /product="hypothetical protein" |
| 5 (581 bp) | complement (34425..35006) | /product="hypothetical protein" |
| 6 (1628 bp) | complement (35242..36870) | /note="COG3378 Predicted ATPase"  /product="putative DNA primase" |
| 7 (794 bp) | complement (36881..37675) | /product="phage DNA replication" |
| 8 (335 bp) | complement (37672..38007) | /product="hypothetical protein" |
| 9 (254 bp) | complement (38004..38258) | /product="hypothetical protein" |
| 10 (239 bp) | complement (38255..38494) | /product="hypothetical protein" |
| 11( 281 bp) | complement (38536..38817) | /product="putative DNA binding protein" |
| 12 (425 bp) | complement (38829..39254) | /product="hypothetical protein" |
| 13 (194 bp) | complement (39259..39453) | /product="hypothetical protein" |
| 14 (185 bp) | complement (39569..39754) | /note="COG1396 Predicted transcriptional regulators"  /product="hypothetical protein" |
| 15 (326 bp) | 39837..40163 | /product="hypothetical protein" |
| 16 (215 bp) | complement (40519..40734) | /product="phage repressor" |
| 17 (440 bp) | 40842..41282 | /note="COG1396 Predicted transcriptional regulators"  /product="phage repressor" |
| 18 (554 bp) | 41312..41866 | /product="hypothetical protein" |
| 19 (230 bp) | 41977..42207 | /product="hypothetical protein" |
| 20 (194 bp) | 42280..42474 | /product="hypothetical protein" |
| 21 (257 bp) | 42727..42984 | /product="hypothetical protein" |
| 22 (1325 bp) | 43098..44423 | /product="hypothetical protein" |
| 23 (206 bp) | 44505..44711 | /product="hypothetical protein" |
| 24 (203 bp) | 44934..45137 | /note="COG4815 Uncharacterized protein conserved in bacteria"  /product="hypothetical protein" |
| 25 (242 bp) | 45201..45443 | /product="prophage ps1 protein 19" |
| 26 (212 bp) | 45591..45803 | /product="Ps108 protein" |
| 27 (1223 bp) | 46467..47690 | /note="COG4584 Transposase and inactivated derivatives"  /product="transposase for insertion sequence element IS712H" |
| 28 (758 bp) | 47702..48460 | /note="COG1484 DNA replication protein"  /product="putative transposase helper protein for IS712H" |
| 29 (326 bp) | 48562..48888 | /product="hypothetical protein" |
| 30 (164 bp) | 49622..49786 | /product="hypothetical protein" |
| 31 (305 bp) | 49908..50213 | /product="hypothetical protein" |
| 32 (1184 bp) | 50463..51647 | /note="COG0582 Integrase"  /product="phage integrase" |
| 33 (713 bp) | complement (52156..52869) | /note="COG4814 Uncharacterized protein with an  alpha/beta hydrolase fold"  /product="hypothetical protein" |
| 34 (365 bp) | 53631..53996 | /product="hypothetical protein" |
| 35 (492 bp) | 54401..54892 | /product="hypothetical protein" |
| 36 (353 bp) | 54930..55283 | /product="hypothetical protein" |
| **The third deleted DNA region (2211286-2229191, 17.9 kb, 34 ORFs)** | | |
| 1 (725 bp) | complement (2210955..2211680) | /note="Derived by automated computational  analysis using gene prediction method: Protein  Homology."  /product="hypothetical protein" |
| 2 (1358 bp) | complement (2211673..2212995) | /note="Derived by automated computational  analysis using gene prediction method: Protein  Homology."  /product="abortive infection protein" |
| 3 (332 bp) | complement (2213442..2213774) | /note="Derived by automated computational  analysis using gene prediction method: Protein  Homology."  /product="hypothetical protein" |
| 4 (440 bp) | complement (2213774..2214214) | /note="Derived by automated computational  analysis using gene prediction method: Protein  Homology."  /product="terminase" |
| 5 (109 bp) | 2214301..2214410 | [repeat region](http://www.ncbi.nlm.nih.gov/nuccore/389853198?from=2214301&to=2214410&sat=4&sat_key=145530746) |
| 6 (542 bp) | complement (2214347..2214889) | /note="Derived by automated computational  analysis using gene prediction method: Protein  Homology."  /product="hypothetical protein |
| 7 (130 bp) | 2214918..2215048 | [repeat region](http://www.ncbi.nlm.nih.gov/nuccore/389853198?from=2214918&to=2215048&sat=4&sat_key=145530746) |
| 8 (1628 bp) | complement (2215106..2216734) | /function="Predicted ATPase"  /note="Derived by automated computational  analysis using gene prediction method: Protein  Homology."  /product="DNA primase" |
| 9 (794 bp) | complement (2216745..2217539) | /note="Derived by automated computational  analysis using gene prediction method: Protein  Homology."  /product="hypothetical protein" |
| 10 (439 bp) | 2217479..2217918 | [repeat region](http://www.ncbi.nlm.nih.gov/nuccore/389853198?from=2217479&to=2217918&sat=4&sat_key=145530746) |
| 11 (335 bp) | complement (2217536..2217871) | /note="Derived by automated computational  analysis using gene prediction method: Protein  Homology."  /product="hypothetical protein" |
| 12( 240 bp) | complement (2217941..2218180) | /note="Derived by automated computational  analysis using gene prediction method: Protein  Homology."  /product="hypothetical protein" |
| 13 (281 bp) | complement (2218218..2218499) | /note="Derived by automated computational  analysis using gene prediction method: Protein  Homology."  /product="hypothetical protein" |
| 14 (203 bp) | complement (2218721..2218924) | /note="Derived by automated computational  analysis using gene prediction method: Protein  Homology."  /product="hypothetical protein" |
| 15 (521 bp) | complement (2218921..2219442) | /note="Derived by automated computational  analysis using gene prediction method: Protein  Homology."  /product="hypothetical protein" |
| 16 (274 bp) | 2219418..2219692 | [repeat region](http://www.ncbi.nlm.nih.gov/nuccore/389853198?from=2219418&to=2219692&sat=4&sat_key=145530746) |
| 17 (665 bp) | complement (2219701..2220366) | /function="Uncharacterized phage-encoded protein"  /note="Derived by automated computational  analysis using gene prediction method: Protein  Homology."  /product="hypothetical protein" |
| 18 (221 bp) | complement (2220529..2220750) | /note="Derived by automated computational  analysis using gene prediction method: Protein  Homology."  /product="transcriptional regulator" |
| 19 (542 bp) | 2220921..2221463 | /function="Predicted transcriptional regulators"  /note="Derived by automated computational  analysis using gene prediction method: Protein  Homology."  /product="transcriptional regulator" |
| 20 (755 bp) | 2221473..2222228 | [repeat region](http://www.ncbi.nlm.nih.gov/nuccore/389853198?from=2221473&to=2222228&sat=4&sat_key=145530746) |
| 21 (230 bp) | 2221685..2221915 | /note="Derived by automated computational  analysis using gene prediction method: Protein  Homology."  /product="hypothetical protein" |
| 22 (194 bp) | 2221988..2222182 | /note="Derived by automated computational  analysis using gene prediction method: Protein  Homology."  /product="hypothetical protein" |
| 23 (734 bp) | 2222364..2223098 | /note="Derived by automated computational  analysis using gene prediction method: Protein  Homology."  /product="hypothetical protein" |
| 24 (185 bp) | 2223304..2223489 | /note="Derived by automated computational  analysis using gene prediction method: Protein  Homology."  /product="hypothetical protein" |
| 25 (185 bp) | 2223489..2223674 | /note="Derived by automated computational  analysis using gene prediction method:  GeneMarkS+."  /product="hypothetical protein" |
| 26 (1064 bp) | 2223676..2224740 | /function="Uncharacterized conserved protein"  /note="Derived by automated computational  analysis using gene prediction method: Protein  Homology."  /product="hypothetical protein" |
| 27 (246 bp) | 2225378..2225624 | [repeat region](http://www.ncbi.nlm.nih.gov/nuccore/389853198?from=2225378&to=2225624&sat=4&sat_key=145530746) |
| 28 (314 bp) | 2225392..2225706 | /note="Derived by automated computational  analysis using gene prediction method: Protein  Homology."  /product="hypothetical protein" |
| 29 (230 bp) | 2225828..2226058 | /note="Derived by automated computational  analysis using gene prediction method: Protein  Homology."  /product="hypothetical protein" |
| 30 (194 bp) | 2226510..2226704 | /note="Derived by automated computational  analysis using gene prediction method: Protein  Homology."  /product="bacteriocin" |
| 31 (167 bp) | 2226948..2227115 | [repeat region](http://www.ncbi.nlm.nih.gov/nuccore/389853198?from=2226948&to=2227115&sat=4&sat_key=145530746) |
| 32 (122 bp) | 2227126..2227248 | [repeat region](http://www.ncbi.nlm.nih.gov/nuccore/389853198?from=2226948&to=2227115&sat=4&sat_key=145530746) |
| 33 (1181 bp) | 2227201..2228382 | /function="Integrase"  /note="Derived by automated computational  analysis using gene prediction method: Protein  Homology."  /product="integrase" |
| 34 (612 bp) | 2228595..2229221 | /function="Uncharacterized protein involved in copper resistance"  /note="Derived by automated computational  analysis using gene prediction method: Protein  Homology."  /product="copper homeostasis protein CutC" |
| **The forth deleted DNA region (2073682-2095306, 21.6 kb, 30 ORFs)** | | |
| 1 (5147 bp) | complement (2073682..2078829) | /note="Derived by automated computational  analysis using gene prediction method: Protein  Homology."  /product="phage tail tape measure protein" |
| 2 (416 bp) | complement (2079052..2079468) | /note="Derived by automated computational  analysis using gene prediction method: Protein  Homology."  /product="hypothetical protein" |
| 3 (593 bp) | complement (2079613..2080206) | /note="Derived by automated computational  analysis using gene prediction method: Protein  Homology."  /product="tail protein" |
| 4 (395 bp) | complement (2080237..2080632) | /note="Derived by automated computational  analysis using gene prediction method: Protein  Homology."  /product="hypothetical protein" |
| 5 (506 bp) | complement (2080629..2081135) | /note="Derived by automated computational  analysis using gene prediction method: Protein  Homology."  /product="hypothetical protein" |
| 6 (350 bp) | complement (2081137..2081487) | /note="Derived by automated computational  analysis using gene prediction method: Protein  Homology."  /product="head-tail joining protein" |
| 7 (323 bp) | complement (2081462..2081785) | /note="Derived by automated computational  analysis using gene prediction method: Protein  Homology."  /product="hypothetical protein" |
| 8 (1214 bp) | complement (2081986..2083200) | /function="Predicted phage phi-C31 gp36 major  capsid-like protein"  /note="Derived by automated computational  analysis using gene prediction method: Protein  Homology."  /product="phage capsid protein" |
| 9 (704 bp) | complement (2083212..2083916) | /note="Derived by automated computational  analysis using gene prediction method: Protein  Homology."  /product="peptidase" |
| 10 (1172 bp) | complement (2083962..2085134) | /function="Phage-related protein"  /note="Derived by automated computational  analysis using gene prediction method: Protein  Homology."  /product="portal protein" |
| 11 (209 bp) | complement (2085137..2085346) | /note="Derived by automated computational  analysis using gene prediction method: Protein  Homology."  /product="head-tail joining protein" |
| 12 (1598 bp) | complement (2085315..2086913) | /function="Phage terminase-like protein, large subunit"  /note="Derived by automated computational  analysis using gene prediction method: Protein  Homology."  /product="terminase" |
| 13 (1326 bp) | 2086949..2088275 | [repeat region](http://www.ncbi.nlm.nih.gov/nuccore/389853198?from=2086949&to=2088275&sat=4&sat_key=145530746) |
| 14 (1175 bp) | complement (2087002..2088177) | /note="Derived by automated computational  analysis using gene prediction method: Protein  Homology."  /product="transposase" |
| 15 (284 bp) | complement (2088262..2088546) | /note="Derived by automated computational  analysis using gene prediction method:  GeneMarkS+."  /product="terminase large subunit" |
| 16 (398 bp) | complement (2088536..2088934) | /function="Phage terminase, small subunit"  /note="Derived by automated computational  analysis using gene prediction method: Protein  Homology."  /product="terminase" |
| 17 (518 bp) | complement (2089116..2089634) | /function="Restriction endonuclease"  /note="Derived by automated computational  analysis using gene prediction method: Protein  Homology."  /product="HNH endonuclease" |
| 18 (290 bp) | complement (2089638..2089928) | /note="Derived by automated computational  analysis using gene prediction method: Protein  Homology."  /product="hypothetical protein" |
| 19 (401 bp) | complement (2090305..2090706) | /note="Derived by automated computational  analysis using gene prediction method: Protein  Homology."  /product="hypothetical protein" |
| 20 (185 bp) | complement (2090789..2090974) | /note="Derived by automated computational  analysis using gene prediction method: Protein  Homology."  /product="hypothetical protein" |
| 21 (308 bp) | complement (2090971..2091279) | /note="Derived by automated computational  analysis using gene prediction method: Protein  Homology."  /product="hypothetical protein" |
| 22 (179 bp) | Complement (2091281..2091460) | /note="Derived by automated computational  analysis using gene prediction method:  GeneMarkS+."  /product="hypothetical protein" |
| 23 (2167 bp) | 2091620..2093787 | [repeat region](http://www.ncbi.nlm.nih.gov/nuccore/389853198?from=2091620&to=2093787&sat=4&sat_key=145530746) |
| 24 (758 bp) | complement (2091711..2092469) | /note="Derived by automated computational  analysis using gene prediction method: Protein  Homology."  /product="ATPase AAA" |
| 25 (1223 bp) | complement (2092481..2093704) | /note="Derived by automated computational  analysis using gene prediction method: Protein  Homology."  /product="integrase" |
| 26 (209 bp) | 2094264..2094473 | /note="Derived by automated computational  analysis using gene prediction method: Protein  Homology."  /product="hypothetical protein" |
| 27 (200 bp) | complement (2094522..2094722) | /note="Derived by automated computational  analysis using gene prediction method: Protein  Homology."  /product="hypothetical protein" |
| 28 (167 bp) | 2094555..2094722 | [repeat region](http://www.ncbi.nlm.nih.gov/nuccore/389853198?from=2094555&to=2094722&sat=4&sat_key=145530746) |
| 29 (230 bp) | complement (2094719..2094949) | /note="Derived by automated computational  analysis using gene prediction method: Protein  Homology."  /product="hypothetical protein" |
| 30 (338 bp) | complement (2094968..2095306) | /note="Derived by automated computational  analysis using gene prediction method: Protein  Homology."  /product="hypothetical protein" |

**Table S2. Primers utilized in this study**

| **Primer** | **Sequence (5’ to 3’)** |
| --- | --- |
| For construction of large-scale gene KO vector | |
| **L1 up -F** | CCGCTCGAGTCTAGGCTTTCGCTCTCCTC |
| **L1 up -R** | CCCATTTAAATAAATTCCAGGATTTGGAGC |
| **L1 down -F** | TCGCGATCGACTTAGATACGTGGAACGCATG |
| **L1 down -R** | GAAGATCTAATGCTTGCTCACTTGCTTAAA |
| **L2 up -F** | CCGCTCGAGGAGTATTTGGGATTAACAATGGTG |
| **L2 up -R** | CCCATTTAAATTGGGTTCTGGTGGGTATCA |
| **L2 down -F** | CCCGAGCTCTTGGTCATACCCTTTACTGCAT |
| **L2 down -R** | GAAGATCTACTCCTGTCAGCTTTTATCGTG |
| **L3 up -F** | CCGCTCGAGTCAAGGCATTGGCTTCCA |
| **L3 up -R** | CCCATTTAAATAAATGAAAAGTTATGTCCTTTAATG |
| **L3 down -F** | CCCGAGCTCTTGACTGGAACAAACTACGTT |
| **L3 down -R** | GAAGATCTCCATATCCTAGCGACTGC |
| **L4 up -F** | CCGCTCGAGGATTTGGTACTTTCCCGTCA |
| **L4 up -R** | CCCATTTAAATCCGCAACTATTAATAAGACAC |
| **L4 down -F** | CCCGAGCTCTATTTGGTAGTCTTGCACGAT |
| **L4 down -R** | GAAGATCTACTAAGCGAATTTGAATCAGT |
| For identification of large-scale gene deletion strain | |
| **P-t1/F** | AAATGGCGTAAGATTTTTTTG |
| **P-t1/R** | AATCTAATCCCTATAAGGAGGAA |
| **P-t2/F** | GGTATCGCAAGTAGTAGTCCT |
| **P-t2/R** | ATGTTTATTGACTGTTTTTGTTG |
| **P-t3/F** | ACCACTTTCTGCTAATACACGG |
| **P-t3/R** | CTTGCCATTCAGGATTTAAT |
| **P-t4/F** | CCGCCATAAACATATACCTT |
| **P-t4/R** | AGATGTAGCACAGATACAAC |
| For construction of pLEB124-P8/*lecC*, pLEB124-P5/*lecC* and pNZ8048-*rfp* plasmids | |
| **P8-F** | CCCAAGCTTTTTTTTAGGACAATTATTTCTCATA |
| **P8-R** | GGAAGATCTTATATCTCTCCATTTCTGCTGCT |
| **P5-F** | CCCAAGCTTGAAAAAGAAAATGTTTTTGTATT |
| **P5-R** | GGAAGATCTTGTAACCGTCCTCCTCACTAC |
| **P*rfp*-F** | CGGGGTACCATGGTTTCAAAAGGTGAAGAAG |
| **P*rfp*-R** | TGCTCTAGATTATTTATATAATTCATCCATACCA |
| For RT-qPCR | |
| ***tufA*-F** | CGGCTCTGATTAAATTCTGA |
| ***tufA*-R** | TTGTAATGCGTGGTGATG |
| **Q-*lecC*-F** | AAGAACTATGGAAACGGTGTT |
| **Q-*lecC*-R** | CAGCATTGCCACCGGTTAG |
| **Q-*rfp*-F** | GCTTCATCAGAAAGAATGTATCC |
| **Q-*rfp*-R** | CAGCTCTTTCATATTGTTCAACA |

**

**

**Figure S1** Verification of deletions with testing primer pairs**.**
